# Anti-biofilm Potential of *Elletaria cardamomum* Essential Oil Against *Escherichia coli* O157:H7 and *Salmonella* Typhimurium JSG 1748

**DOI:** 10.3389/fmicb.2021.620227

**Published:** 2021-04-09

**Authors:** Abdullah, Ali Asghar, Ammar Algburi, Qingrong Huang, Talha Ahmad, Hao Zhong, Hafiz U. Javed, Alexey M. Ermakov, Michael L. Chikindas

**Affiliations:** ^1^College of Biosystems Engineering and Food Science, Zhejiang University, Hangzhou, China; ^2^Department of Food Science, Rutgers State University, New Brunswick, NJ, United States; ^3^National Institute of Food Science and Technology, University of Agriculture Faisalabad, Faisalabad, Pakistan; ^4^Department of Biotechnology, College of Science, University of Diyala, Baqubah, Iraq; ^5^Department of Plant Sciences, School of Agriculture and Biology, Shanghai Jiao Tong University, Shanghai, China; ^6^Center for Agrobiotechnology, Don State Technical University, Rostov-on-Don, Russia; ^7^Health Promoting Naturals Laboratory, School of Environmental and Biological Sciences, Rutgers State University, New Brunswick, NJ, United States; ^8^Department of General Hygiene, I.M. Sechenov First Moscow State Medical University, Moscow, Russia

**Keywords:** green cardamom, bioactive compounds, GC-MS characterization, anti-biofilm potential, *Escherichia coli* O157:H7, *Salmonella* Typhimurium JSG 1748, mutagenic activity

## Abstract

Foodborne pathogens, microbial recurrent infections, and antibiotic resistance have driven researchers to explore natural compounds as safe alternative antimicrobials. In this study, the chemical profile, antimicrobial, and mutagenic activities of the *Elletaria cardamomum* essential oil were investigated. GC-MS analysis identified the major bioactive components as α-terpinyl acetate, 1,8-cineole, linalool acetate, and sabinene, at concentrations of 34.95, 25.30, 8.13, and 5.48% respectively, of the essential oil’s content. Regarding antimicrobial activity, the minimum inhibitory concentration of green cardamom essential oil was 1% against *Escherichia coli* O157:H7 and *Pseudomonas aeruginosa* ATCC 14213. Green cardamom essential oil, when used at concentrations of 0.015, 0.031, 0.062, and 0.125% (v/v) prevented biofilm formation of *Escherichia coli* O157:H7 by 64.29, 65.98, 70.41, and 85.59%, respectively. Furthermore, these concentrations inhibited 6.13, 45.50, 49.45, and 100%, respectively, of the *Salmonella* Typhimurium JSG 1748 biofilm. A mutagenicity assay confirmed that green cardamom essential oil has no demonstrable mutagenic activity against the tested strains. The study’s findings suggest that green cardamom derived bioactive compounds are safe organic antimicrobials, effective in controlling biofilm formation by Gram-negative pathogens. Moreover, such compounds could possibly be used in the food industry (e.g., bakery, dairy, meat, and other food products) as a safe alternative to chemical preservatives (antimicrobials) to enhance shelf life by improving the antimicrobial status while at the same time imparting a pleasant and appealing aroma for consumers.

## Introduction

Interest in the application of bioactive phytochemicals and essential oils in food and pharmaceutical products has rapidly increased due to their health benefits, including their antioxidant, antimicrobial, and hypolipidemic properties (Deepa et al., 2013; Aghasi et al., 2019; Yousefi et al., 2019; Abdullah et al., 2020; Dehghani et al., 2020). A wide variety of herbs and spices have been used in cooking and medicine, particularly in the treatment of gastrointestinal disorders, since ancient times. Many studies have reported the health benefits of cardamom derived bioactive phytochemicals related to their antimicrobial (e.g., bacterial growth, biofilm, and quorum sensing inhibition), gastro-protective (anti-ulcer), hypocholesterolemic, and hypotensive activities (Verma et al., 2009; Sharma et al., 2011; Abdullah et al., 2017, 2020; Chakraborty et al., 2019).

*Elletaria cardamomum*, also called green or true cardamom, is a member of the family *Zingiberaceae*. Green cardamom is used as a functional additive in food products due to its pleasant aroma, as a flavor enhancer, and as a preservative in confectionery, beverages, bakery, dairy, and meat products. The antioxidant and antibacterial potentials of green cardamom are due to phenolic and flavonoid compounds present in the spice (Padmakumari Amma et al., 2010; Abdullah et al., 2017). For the extraction of the bioactive compounds present in the green cardamom, various techniques have been used such as conventional solvent extraction, hydro-distillation, and super-critical fluid extraction (SFE; Azmir et al., 2013; Yousefi et al., 2019). In recent years, the extraction of phytochemicals and essential oils-comprising bioactive compounds by SFE has gained a greater level of attention and is now the most widely-used method due to reduced sample degradation, higher solubilization, improved processing time and technological selectivity (e.g., temperature, pressure, and extraction time), no requirement for clean-up steps, and its eco-friendly nature (Yousefi et al., 2019). Moreover, the extraction efficiency, time, temperature, and pressure conditions can be adjusted in the SFE system according to the targeted analytes (Marongiu et al., 2004; Junior et al., 2010).

A bacterial biofilm is a three-dimensional structure, formed by mono- or multi-microbial communities embedded into an extracellular matrix, which provides resistance to microorganisms against harsh environmental conditions, antibiotics, and the defenses of the human immune system (Miquel et al., 2016; Jamal et al., 2018). Recently, Nassar et al. (2021) reported that microorganisms responsible for various human infections (~80%) and hospital-acquired infections (60–70%), have shown a biofilm origin. Therefore, to eradicate biofilm-related persistent infections and to reduce their impact on human health, it is necessary to find safe antimicrobials as anti-biofilm agents, which can inhibit biofilm formation. Furthermore, the emergence of antibiotic resistant pathogens is a key factor driving the need for novel alternative antimicrobial compounds with the ability to inhibit biofilm formation, as biofilms play an important role in the development of antimicrobial resistance (emergence and dissemination), and their persistence may eventually lead to the failure of currently available treatments for many bacterial and other species infections (Livermore, 2011; Nassar et al., 2021). Bacterial strains are becoming increasingly resistant to conventional antibiotics; therefore, safe and effective antimicrobial agents are urgently required to counteract multidrug-resistant microorganisms (Newman and Cragg, 2016). In this regard, many herbs and plants represent a promising source of biologically active compounds with antimicrobial properties (anti-biofilm) and with limited negative side effects on human health (Newman and Cragg, 2016; Abdullah et al., 2020). In recent years, some studies established the antimicrobial potential of cardamom-derived bioactive compounds against *Escherichia coli*, *Streptococcus mutans, Salmonella* Typhimurium, *Staphylococcus aureus*, and *Candida albicans* (Aneja and Joshi, 2009; Abdullah et al., 2017, 2020).

Controlling biofilm formation by pathogenic bacteria remains a challenging issue that requires the discovery and analysis of effective and safe alternative antimicrobials that may be used for the prevention of antibiotic resistance and infection recurrence. This study aimed to investigate the green cardamom essential oil (GCEO) chemical profile (bioactive components), antimicrobial, and mutagenic properties with the following objectives; (i) quantification of GCEO bioactive compounds using GC-MS, (ii) determination of the minimum inhibitory concentrations (MICs), and biofilm inhibitory concentrations of GCEO against *Escherichia coli* O157:H7 and *Salmonella* Typhimurium JSG 1748, and (iii) investigating the mutagenic activities of GCEO using *Salmonella* Typhimurium TA98 and *Salmonella* Typhimurium TA100 strains.

## Materials and Methods

### Chemical Reagents and Materials

Green cardamom was procured (Faisalabad, Punjab, Pakistan), cleaned, and milled into powder. All reagents used in this study were of HPLC grade and acquired from Sigma (Sigma-Aldrich, Tokyo, Japan) and Merck (Merck KGaA, Darmstadt, Germany). Tryptic soy agar (TSA, Thermo Fisher Scientific Remel Products, Lenexa, KS, United States), and tryptic soy broth (TSB, BD Difco, Franklin Lakes, NJ, United States) were used to grow and maintain the bacterial strains. The Muta-ChromoPlate™ Bacterial Strain Kit, a 96-well microplate, was purchased and used for detection of mutagenic activity, and TA100 and TA98 were the default bacterial strains included in the Muta-ChromoPlate kit (Muta-ChromoPlate™ Bacterial Strain Kit: Product No. B5051, Environmental Bio-Detection Products Inc. (EBPI), Mississauga, Canada).

### Supercritical Fluid Extraction of GCEO

GCEO was obtained using a supercritical fluid extraction (SFE) instrument (model SFT-150, Applied Separations, Inc., Allentown, PA, United States) using 99.8% pure CO_2_ at 30°C, 300 bar, and 60 min according to Abdullah et al. (2020). The sample (100 g) in powder form was loaded into the extraction vessel, and the gas (CO_2_) was liquefied by employing the SFE conditions (temperature, pressure, and time), which facilitated mass transfer in the form of green cardamom essential oil (GCEO). GCEO was collected in a vial and kept in the refrigerator at 4°C for further analyses.

### GC-MS Analysis of GCEO

The GCEO sample (10 μl) was diluted in per ml of tertiary butyl methyl ether and examined using GC-MS according to the guidelines of Adams (2017) with minor modifications. The diluted GCEO sample (1 μl) was injected into the column (SH-RXI-5SIl MS, 30 m × 0.25 mm × 0.25 μm) by an auto sampler (AOC 6000). The GC-MS analysis of the sample was carried out under the following conditions: the initial temperature was 35°C for 4 min, which reached up to 250°C by increasing at a rate of 20°C per min, and the flow rate of helium (carrier gas) was set as constant (1 ml per min). The ionization was performed in the electron impact mode at 230°C and an ionization energy of 70 eV. Mass spectra were obtained in full scan mode (mass range *m/z* 35-450) under auto-tuning conditions. The identification of bioactive compounds was carried out by matching the spectra with the mass spectral libraries, and the identity of each compound was confirmed by comparing its Kovat’s index with the libraries (Wiley275.L) and compounds from the literature (Adams, 2017). Table 1 shows the isolated GCEO bioactive compounds listed in order of elution from the SH-RXI-5Sil MS column, and their relative percentage was computed from the chromatogram peak area by the peak integration method using a MS detector.

**Table 1:** GC-MS quantification of *Elletaria cardamomum* bioactive compounds.

Peak No.	Retention time (min)	Bioactive compounds	Composition (%^*^)
1	7.619	a-Thujene	0.20
2	7.719	a-Pinene	1.81
3	8.176	Sabinene	5.48
4	8.244	b-Pinene	0.36
5	8.328	Myrcene	1.76
6	8.720	p-Cymene	0.14
7	8.777	Limonene	2.80
8	8.817	1,8-Cineole	25.30
9	9.048	g-Terpinene	0.12
10	9.169	Linalool oxide	0.15
11	9.397	Terpinolene	2.30
12	9.459	Linalool	1.87
13	10.152	Tetrahydro Linalool	0.19
14	10.259	a-Terpineol	2.79
15	10.405	cis-Sabinene hydrate acetate	1.02
16	10.557	Geraniol	0.24
17	10.609	Linalool acetate	8.13
18	10.663	Unknown	0.11
19	10.771	Geranial	0.45
20	11.143	Acetate	0.15
21	11.386	a-Terpinyl acetate	34.95
22	11.525	Geranyl acetate	1.02
23	11.972	g-Elemene	0.11
24	12.449	a-Farnesene	0.54
25	12.763	(E) Nerolidol	1.57
26	12.831	Unknown	0.15
27	14.942	n-Hexadecanoic acid	0.79
28	15.999	Fatty acids (C18)	3.27
29	16.167	Fatty acids (C18)	0.25
30	17.187	Fatty acids (C18)	0.28
		Total	98.39

* %, Relative percentage of the bioactive compounds computed from the chromatogram peak area.

### Microbial Strains and Growth Conditions

*Escherichia coli* O157:H7, *Pseudomonas aeruginosa* ATCC 14213, and *Salmonella* Typhimurium JSG 1748 were used as tested strains in this study. The bacterial strains were selected as representative of Gram-negative pathogens, responsible for foodborne diseases and also have medical importance. Tryptic soy broth (TSB) was used for the growth and maintenance of the bacterial strains under aerobic condition for 20–24 h with continuous shaking (200 rpm) at 37°C. Tryptic soy agar was used for bacterial plating and enumeration.

### Evaluation of Minimum Inhibitory Concentrations of GCEO

A broth micro-dilution assay was performed to determine the MIC value of the GCEO according to Algburi et al. (2017), with minor modifications. Briefly, the frozen stocks of the tested strains were grown in TSB media and incubated aerobically at 37°C for 20–24 h to obtain ~10^9^ CFU ml^−1^. The spot plate method was used to confirm the bacterial cell count, then the overnight grown bacterial cultures were diluted (1:1000, v:v) in fresh TSB media to achieve bacterial cell count ~10^6^ CFU ml^−1^. GCEO was first dissolved in ethanol (95%) to prepare a 100 mg ml^−1^ stock solution and then further diluted into TSB media. The dilutions were prepared ranging from 0.015–2% (v/v) using a 96-well micro-plate. Subsequently, 100 μl of the diluted bacteria cells (10^6^ CFU ml^−1^) were added separately into each well of a 96-well micro-plate which had previously been treated with a certain concentration of GCEO. As a control, ethanol was separately diluted with TSB broth (0.1425–1.9%) and evaluated against the growth of the tested bacterial strains. The results showed that the above-mentioned concentrations of ethanol (used as control) had no antibacterial activity. Mineral oil (70 μl) was pipetted into each well of the micro-plate to avoid evaporation of the mixture during the incubation time. The micro-plate was incubated aerobically for 24 h at 37°C inside the plate reader (SmartSpec™ 3000, Bio-Rad, Hercules, CA, United States and ThermoMax, Molecular Devices, San Jose, CA, United States). MIC values of the GCEO were calculated from the kinetic growth curve readings after incubation at an optical density (OD 595 nm). The kinetic curves of bacterial growth were drawn statistically, and the experiments were repeated at least twice and in duplicate. “The MIC is defined according to CLSI (Clinical and Laboratory Standards Institute, 2010) guidance as the lowest concentration of an antimicrobial substance that inhibits bacterial growth in the wells with an OD 595 nm equal to or less than 20% of the control’s mean absorbance (bacterial growth without antimicrobial addition). The MIC was determined based on the tested concentration at which a kinetic growth curve reading showed little to no increase in cell density during the 24 h incubation time indicating bacterial growth inhibition. The non-inhibitory concentrations (NICs) of GCEO were defined as the concentrations that had no measurable effect on the kinetic growth curve readings.” While the minimum bactericidal concentration (MBC), which is an option to be determined, is defined as the lowest concentration of an antimicrobial that completely kills the bacterial cells, in which the OD 595 nm of treated wells will be equal to OD of the negative control (broth only).

### Evaluation of Biofilm Formation Inhibition by GCEO

The biofilm inhibitory effect of GCEO was evaluated against *E. coli* O157:H7 and *S*. Typhimurium JSG 1748. The biofilm formation inhibition analysis was performed according to Algburi et al. (2020). Briefly, *E. coli* and *S*. Typhimurium were grown aerobically using Luria Bertani (LB broth), (BD Difco, Fisher Scientific UK Ltd., Leicestershire, England) supplemented with 1% of glucose (LBG) for 24 h at 37°C. GCEO was diluted ranging from 0.007–0.125% (v/v) into a 96-well micro-plate with a final volume of 100 μl. The overnight grown bacteria were diluted (1:40) in fresh LBG culture medium and 100 μl of the diluted suspension was transferred into each well of the 96-well micro-plate which was pre-treated with GCEO and incubated for 48 h at 37°C. After incubation, the planktonic cells were carefully aspirated and enumerated using the spot plate method and the wells of the plate were washed twice with fresh culture media after incubation. The plate was then heat-fixed for 60 min at 60°C. A sample of 125 μl of crystal violet (0.1%) was added into each well and the plate was further incubated for 20 min at room temperature. The plate was then washed with sterile water three to four times. Afterwards, 200 μl ethanol (95%) was added into each well and the plate was incubated for 30 min at 4°C. Finally, 100 μl of the solubilized crystal violet was transferred into a new 96-well micro-titer plate and the absorbance was measured using a plate reader (SmartSpec™ 3000) at 595 nm. The experiment was conducted three times in duplicate.

### Evaluation of GCEO Mutagenicity Using Ames Test

*S*. Typhimurium TA98 and TA100 strains were selected as representative mutant strains to assess the mutagenic activity of GCEO by the Ames test using a micro-titer plate method (Nighat and Shahid, 2017). Standard mutagens, i.e., potassium dichromate (30 μg 100 μl^−1^) for *S*. Typhimurium TA98 and sodium azide (0.5 μg 100 μl^−1^) for *S*. Typhimurium TA100 were used. Briefly, the strains were initially grown and maintained in nutrient culture media and incubated aerobically for 18–24 h at 37°C. The reaction mixture was carefully prepared using DM salt (21.62 ml), D-glucose (4.75 ml), bromocresol purple (2.38 ml), D-biotin (1.19 ml), and L-histidine (0.06 ml). Then 2.5 ml reaction mixture solution was transferred into a sterilized petri plate along with 17.5 ml deionized distilled water, 0.05 ml of the tested sample and 0.05 ml of the tested strains. The solution in the petri plate was thoroughly mixed and then 150 μl poured into each well of a 96-well plate. To check for mutagenicity, 96-well plates were wrapped with aluminum foil, tightly sealed in plastic bags, and incubated for 3 days at 30°C. The results were expressed based on the color observation (Supplementary Figure S1). After the incubation period, the blank 96-well plate with purple color indicated no contamination. At the time of evaluation; background, and test plates were considered positive (mutagenic) when yellow, partial yellow, and turbid colors were produced, while a purple color was reported as being negative (non-mutagenic). GCEO is considered toxic towards tested strains when all wells of the 96-well plate exhibited a purple color. However, for GCEO to be mutagenic, the number of positive wells had to be considerably higher compared to the number of positive wells in the ‘background’ (negative control) plate which is representative of spontaneous mutations.

### Statistical Analysis

Experiments were carried out at least three times in duplicate. All calculations were made in Microsoft Excel. The experimental data was shaped using SigmaPlot 11.0 (Systat Software Inc., Chicago, IL, United States).

## Results

### GC-MS Quantification of the Bioactive Compounds in GCEO

**Figure 1:**
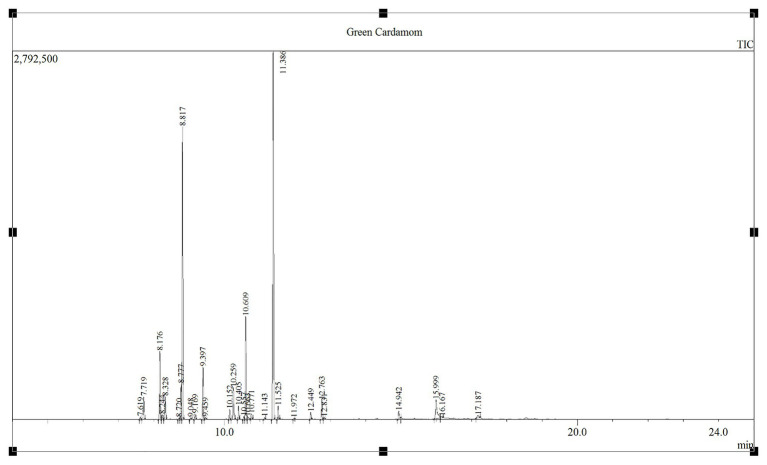
The chromatogram elaborating GCEO bioactive compounds analyzed by the GC-MS (Shimadzu GC 2010 Plus and GCMS-TQ8040) through Shimadzu SH-Rxi-5Sil MS column (30 m long, 0.25 mm ID, 0.25 μm coated film).

The GC-MS analysis of GCEO identified a total of 30 bioactive compounds including; α-terpinyl acetate, 1,8-cineole, linalool acetate, and sabinene which comprised 34.95, 25.30, 8.13, and 5.48% of the identified compounds, respectively. In addition, several other natural compounds were reported which include; g-elemene, α-farnesene, α-thujene, α-pinene, β-pinene, p-cymene, α-terpineol, limonene, linalool oxide, cis-sabinene hydrate acetate, geranyl acetate, geranial, geraniol, myrcene, nerolidol, and n-hexadecanoic acid (Figure 1; Table 1).

### Minimum Inhibitory Concentrations of GCEO Against Tested Gram-Negative Bacteria

**Figure 2:**
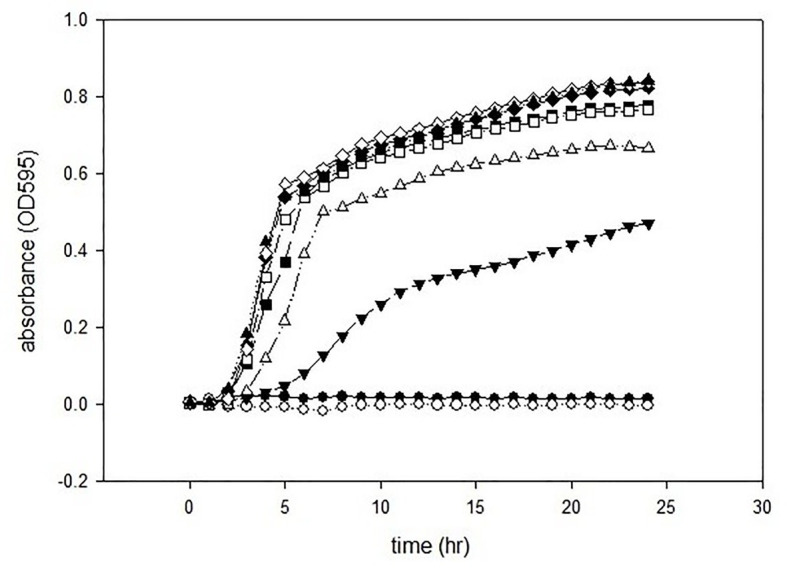
Minimum inhibitory concentrations of GCEO against *Escherichia coli* O157:H7. GCEO used at 2% (●), 1% (○), 0.5% (▼), 0.25% (∆), 0.0125% (■), 0.063% (□), 0.031% (♦), 0.015% (◊), and positive control (▲).

**Figure 3:**
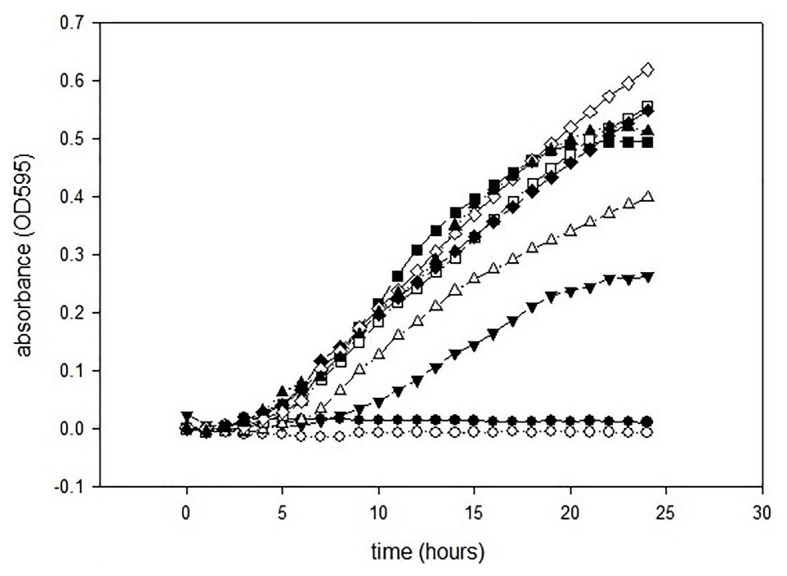
Minimum inhibitory concentrations of GCEO against *Pseudomona aeruginosa* ATCC 14213. GCEO used at 2% (●), 1% (○), 0.5% (▼), 0.25% (∆), 0.0125% (■), 0.063% (□), 0.031% (♦), 0.015% (◊), and positive control (▲).

The antimicrobial activities of GCEO were assessed against selected Gram-negative pathogens. The MIC value of the green cardamom essential oil was 1% against *E. coli* O157:H7 and *P. aeruginosa* ATCC 14213 (Figures 2, 3). These findings indicate that GCEO was an effective antimicrobial substance against the Gram-negative bacteria and has the potential to be used in formulating novel organic anti-infective drugs to counteract multidrug resistant microorganisms. Due to its safety profile, GCEO can be considered as a potent natural formulation preventing biofilm formation by foodborne pathogens. Moreover, GCEO can also be used as an alternative to chemical preservatives (antimicrobials) in the food industry to enhance the shelf life of food products by improving the antimicrobial status while at the same time imparting a pleasant and appealing aroma to bakery, dairy, meat, and other food products.

### Biofilm Inhibitory Effect of GCEO Against Tested Bacteria

**Figure 4:**
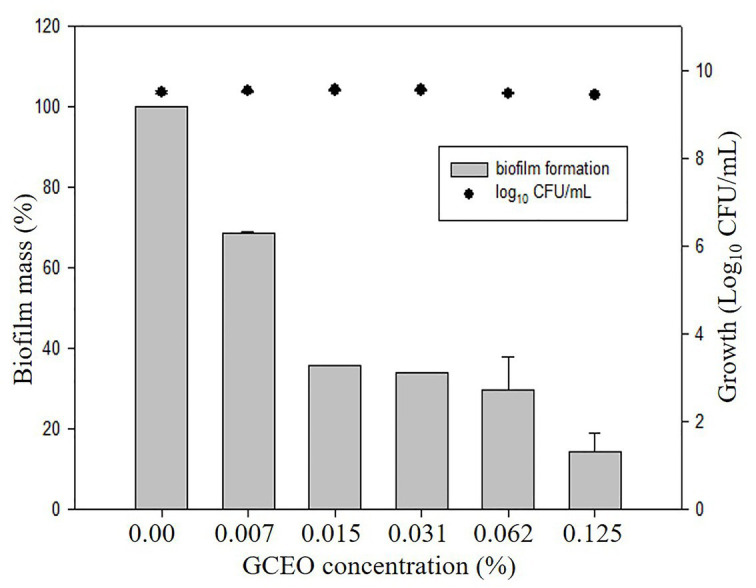
Anti-biofilm activity of GCEO against planktonic and biofilm associated cells of *Escherichia coli* O157:H7. The columns (█) refer to the biofilm mass (%). The solid closed circles (●) refer to the growth (number) of planktonic cells (Log_10_ CFU ml^−1^) vs. untreated cells.

**Figure 5:**
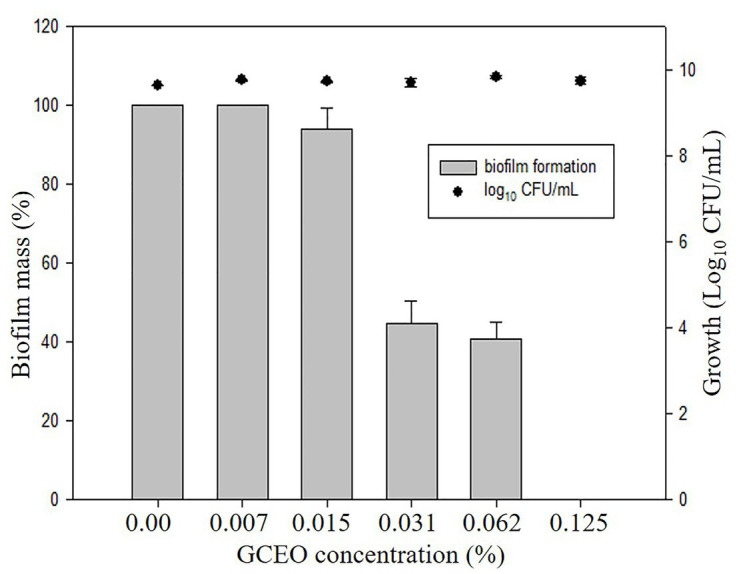
Anti-biofilm activity of GCEO against planktonic and biofilm associated cells of *Salmonella* Typhimurium JSG 1748. The columns (█) refer to the biofilm mass (%). The solid closed circles (●) refer to the growth (number) of planktonic cells (Log_10_ CFU ml^−1^) vs. untreated cells.

Bioactive compounds and essential oils derived from aromatic herbs, spices, and plants are considered as safe and promising alternative antimicrobial therapies. *E. coli*, *S*. Typhimurium, and *P. aeruginosa* were used to evaluate the GCEO activity against planktonic cells of the selected Gram-negative pathogens, while *E. coli* and *S*. Typhimurium were used to investigate the biofilm inhibitory activity of the cardamom derived bioactive compounds. Regarding the anti-biofilm activity of GCEO, *E. coli* and *S*. Typhimurium biofilms were found to be inhibited at sub-MIC concentrations. When GCEO at concentrations of 0.007, 0.015, 0.031, 0.062, and 0.125% (v/v) were used, 31.25, 64.29, 65.98, 70.41, and 85.59% of *E. coli* O157:H7 biofilm formation was inhibited, respectively (Table 2). Likewise, the same concentrations of GCEO (0.007, 0.015, 0.031, 0.062, and 0.125%, v/v) prevented 0.0, 6.13, 45.50, 49.45, and 100% of biofilm formation by *S*. Typhimurium. Furthermore, we noticed that the growth of the planktonic cells of *E. coli* and *S*. Typhimurium were not influenced by the tested concentrations (Table 2), which indicates that GCEO may be acting on the virulence factor(s) regulating biofilm formation, without interrupting the bacterial growth *via* direct antimicrobial action (Figures 4, 5).

**Table 2:** Inhibition of biofilm formation by *Escherichia coli* O157:H7 and *Salmonella* Typhimurium JSG 1748 using GCEO.

Bacterial strain	Concentration (%)	Biofilm mass (%)	Inhibition (%)	Log_10_ CFU ml^−1^
*E. coli*	0.000	100 ± 0.00	-	9.50 ± 0.03
	0.007	68.75 ± 0.07	31.25	9.52 ± 0.02
	0.015	35.71 ± 0.00	64.29	9.55 ± 0.01
	0.031	34.02 ± 0.00	65.98	9.55 ± 0.02
	0.062	29.59 ± 8.32	70.41	9.48 ± 0.02
	0.125	14.41 ± 4.41	85.59	9.43 ± 0.00
*S*. Typhimurium	0.000	100 ± 0.00	-	9.62 ± 0.01
	0.007	100 ± 0.00	-	9.75 ± 0.04
	0.015	93.87 ± 5.44	6.13	9.72 ± 0.02
	0.031	44.50 ± 5.69	45.50	9.69 ± 0.08
	0.062	40.55 ± 4.48	49.45	9.83 ± 0.03
	0.125	0.00 ± 0.00	100	9.74 ± 0.09

### GCEO Mutagenic Activity

Any substance or bioactive compound could be appraised as mutagenic if “the number of the positive-wells with the tested sample are more than twice the positive-wells in the negative control plate (background) i.e., induced mutation by standard mutagen” (Nighat and Shahid, 2017). GCEO was found to be non-mutagenic by this definition, as there were no significant results related to the mutagenicity of GCEO in the *S*. Typhimurium TA98 and TA100 bacterial strains (Table 3; Supplementary Figure S1). Thus, GCEO use is suggested in food and pharmaceutical applications as an alternative to chemical preservatives and conventional anti-infective drugs due to its safety for human health, which was confirmed in previously published reports (Al-Othman et al., 2006) and by Ames test-based evaluation of its mutagenic activity.

**Table 3:** The mutagenic activities of green cardamom essential oil.

*S*. Typhimurium TA98			*S*. Typhimurium TA100	
Sample	No. of +ive wells/No. of total wells	Results	No. of +ive wells/No. of total wells	Results
Background	14/96	-	27/96	-
Cardamom + ethanol	24/96	Non-mutagenic	2/96	Non-mutagenic
Cardamom	18/96	Non-mutagenic	10/96	Non-mutagenic

## Discussion

Many studies have reported on the health benefits of herbs and plants including different cardamom cultivars in terms of their antioxidant, antimicrobial, and hypolipidemic potentials and potential for food applications such as functional additives (Deepa et al., 2013; Abdullah et al., 2017, 2020; Aghasi et al., 2019; Dehghani et al., 2020). In the healthcare and pharmaceutical industries, interest in plant-derived bioactive compounds (phytochemicals) as potential medicines has attracted the attention of researchers due to their health-promoting characteristics, such as their antioxidant, antimicrobial, anti-ulcer (gastro-protective), anti-thrombotic, and anti-hypertensive effects (Verma et al., 2009; Sharma et al., 2011; Chakraborty et al., 2019). Specifically, bioactive compounds derived from different cardamom cultivars have shown promising antimicrobial activity against Gram-positive and Gram-negative pathogens (Abdullah et al., 2017, 2020). The present study investigated several key characteristics of the GCEO, including the chemical profile, antimicrobial properties (growth and biofilm inhibitory effects), and potential mutagenic activity to determine its potential use as a safe organic antimicrobial substance.

### GC-MS Quantification of the Bioactive Compounds in GCEO

GC-MS analysis showed that α-terpinyl acetate and 1,8-cineole were the principle bioactive compounds in GCEO. Cardamom of Indian origin contains 1,8-cineole (36.30%) and α-terpinyl acetate (31.30%) as major compounds, however, the percentages of 1,8-cineole and α-terpinyl acetate varies between 26.50–63.30% and 23.20–52.50%, respectively (Parthasarathy et al., 2008). Our results were in agreement with previous findings published by Aneja and Joshi (2009) who reported that the major constituents of green cardamom include α-terpinyl acetate, 1,8-cineole, α-terpineol, and linalool at 44.3, 10.7, 9.8, and 8.6%, respectively. Likewise, Nair (2006) reported a wide range of concentrations of 1,8-cineole (27–36.1%) and α-terpinyl acetate (38.5–47.9%) as the major volatile compounds in the GC-MS analysis of cardamom extracts. A cardamom seed oil obtained by supercritical extraction using CO_2_ as a solvent was found to have 42.3% α-terpinyl acetate, 21.40% 1,8-cineole, 8.2% linalyl acetate, 5.6% limonene, and 5.4% linalool as major constituents (Marongiu et al., 2004). The GC-MS results of the present study were in line with the findings of Savan and Kucukbay (2013) who identified α-terpinyl acetate, 1,8-cineole, and linalool as major compounds at 40.7, 25.6, and 6.3%, respectively. The variations between the present findings and earlier studies regarding the chemical composition of GCEO could be due to several factors including the origin of the cardamom, extraction techniques, and variations in experimental conditions, specifically the conditions used for analytical chromatography and the stationary phase being used in the column.

### GCEO Antimicrobial Perspectives

Nowadays, *E. coli* is a matter of great concern to the food industry because it can produce toxins in food commodities that ultimately result in food toxicity and food poisoning. Moreover, it is equally important to the pharmaceutical industry, as *E. coli* is also responsible for urinary tract infections (~90%) and other serious harmful effects are also associated with its presence inside the human body. Many bioactive phytochemicals possess promising antimicrobial potential and may be used as organic antimicrobial agents for food and pharmaceutical applications (Yousefi et al., 2019; Abdullah et al., 2020).

Bioactive phytochemicals play an important role in the survival of producing species as part of their defense system against pathogens. Plant-derived bioactive compounds that exhibit antimicrobial properties are most often flavonoids, phenolics, quinines, saponins, tannins, coumarins, terpenoids, and alkaloids (Choo et al., 2003; Mendez-Vilas, 2013; Nighat and Shahid, 2017). Recently, a group of researchers described the antimicrobial activities of a flavonoid extract of pummel peel; the MIC value of the extract ranged from 0.5 to 4.5 mg ml^−1^ against the *Vibrio anguillarum* and *Chromobacterium violaceum* CV026 bacterial strains (Liu et al., 2017). Likewise, Tapia-Rodriguez et al. (2017) studied the antimicrobial potential and identified the minimal inhibitory concentrations (MIC) and the minimal biofilm inhibitory concentrations (MIC-B) of carvacrol against *P. aeruginosa*. The MIC value of carvacrol was 7.9 mM against *P. aeruginosa* while biofilm formation inhibition was observed at sub-MIC values. Bahari et al. (2017) investigated the synergistic effect of curcumin in combination with antibiotics (azithromycin and gentamycin) against *P. aeruginosa*. The authors reported a reduction in the MICs of azithromycin and gentamycin when they were combined with curcumin. Similarly, McCarthy and Ogara (2015) found that garlic (*Allium sativum*) extract synergized with tobramycin to kill *P. aeruginosa*. They concluded that garlic extract not only decrease the pathogenicity of bacteria but also increased microbial susceptibility to antibiotics.

Sheng et al. (2016) explored the antimicrobial effect of grape seed extract on the generation of toxins by *E. coli*, a matter of great concern to researchers due to the harmful effects to consumers related to the expression of virulence factors in *E. coli*. This study reported that 4 mg ml^−1^ of grape seed extract inhibited the growth of *E. coli*. Recently, Amrutha et al. (2017) found that organic acids obtained from fresh fruits and vegetables have potential for use as antimicrobials. They investigated the effects of different acids such as citric acid, acetic acid, and lactic acid on biofilm development and as anti-quorum sensing compounds. They observed 39% biofilm formation inhibition with lactic acid for *E. coli* and 22% with citric acid for *Salmonella*. They also applied a lactic acid solution (2%) on cucumbers and found it to be very effective in inhibiting the growth of *E. coli* and *Salmonella*. They recommended the use of these natural acids as efficient disinfectants and natural antimicrobials to inhibit bacterial growth on fresh fruits and vegetables. *E. coli* is the most common pathogen of the urinary tract and is isolated in 90% of cases of individuals with urinary tract infections. A group of researchers explored the ability of the *Melia dubia* plant derived compound (fructose furoic acid ester) to down regulate the virulence expression of virulence factors and found it effective as an inhibitor to combat uropathogen *E. coli* biofilm formation (Vinothkannan et al., 2018).

Vasavi et al. (2016) investigated the antimicrobial activity of an extract of the traditional herb *Centella asiatca* and its impact on the biofilm formation inhibition of *P. aeruginosa* and found that the extract had antimicrobial activity against bacterial biofilms. Several studies have reported that plant-derived bioactive compounds inhibited quorum sensing by hindering synthesis of peptidoglycan, interruption in membrane structures, or inhibitory effects on signal detection and subsequently inhibit biofilm formation. A study reported that 4 mg ml^−1^ of cardamom extract obtained through conventional solvent extraction prevented biofilm formation in *S*. Typhimurium (51.96%), and *P. aeruginosa* (45.28%) (Niu and Gilbert, 2004; Rahman et al., 2017). Likewise, *Cinnamomum verum*, *Juniperus communis*, *Origanum majorana*, *Medicago sativa*, *Syzygium aromaticum*, and *Salvia sclarea* essential oils exhibited antimicrobial activities by preventing biofilm formation (Kerekes et al., 2013; Soni et al., 2013; Newman and Cragg, 2016). Previously, ginger based phenolic derivatives showed inhibitory effects. The compounds zingerone and gingerol revealed 35 and 50% quorum sensing inhibition, respectively, at 500 ppm against *C. violaceum*, a Gram-negative bacterium (Kumar et al., 2014). The bacteriostatic properties of GCEO observed in the present study against the tested bacterial strains, provide scientific justification as a promising antimicrobial treatment. However, more studies are required regarding the safety, purification, and isolation of cardamom’s bioactive compounds as anti-infective agents, to better understand their mechanisms of action in controlling microbial virulence factors and in the development of anti-biofilm therapies. Compared to the extract, the purified compounds, certainly, will possess a higher potency against pathogens, and reduce bacterial resistance to antimicrobial agents.

## Conclusion

GC-MS analysis identified 30 bioactive compounds. Among them, α-terpinyl acetate (34.95%) and 1,8-cineole (25.30%) were present as the principal compounds in GCEO. A minimum inhibitory concentration assay revealed that a GCEO concentration of 1% inhibited the growth of *Escherichia coli* O157:H7 and *Pseudomonas aeruginosa* ATCC 14213. Anti-biofilm evaluation showed that the tested concentrations (0.015, 0.031, 0.062, and 0.125%, v/v) caused 64.29, 65.98, 70.41, and 85.59% biofilm formation inhibition in *Escherichia coli* and 6.13, 45.50, 49.45, and 100% inhibition of *Salmonella* Typhimurium, respectively. The mutagenicity assay confirmed GCEO non-mutagenic potential against *Salmonella* Typhimurium TA98 and *Salmonella* Typhimurium TA100 strains. Based on these findings, green cardamom essential oil could possibly be used as a safe antimicrobial (organic substance) to inhibit microbial biofilms and counteract multidrug resistant microorganisms. The food industry can also use green cardamom-derived bioactive compounds as safe alternatives to chemical preservatives (antimicrobials) to develop food products with an enhanced shelf life and a pleasant and appealing aroma for consumers.

## Data Availability Statement

The raw data supporting the conclusions of this article will be made available by the authors, without undue reservation.

## Author Contributions

Abdullah, AAl, and QH: conceptualization. Abdullah, AAl, and MC: methodology, investigation, writing, review and editing. Abdullah, TA, and HJ: writing of original draft preparation. AAs and QH: supervision. Abdullah, MC, and AE: resources and funding acquisition. All authors contributed to the article and approved the submitted version.

### Conflict of Interest

The authors declare that the research was conducted in the absence of any commercial or financial relationships that could be construed as a potential conflict of interest.
