# Supplementary figures and images for: Anti-biofilm Potential of Elletaria cardamomum Essential Oil Against Escherichia coli O157:H7 and Salmonella Typhimurium JSG 1748
Source: Front Microbiol. 2021 Apr 9;12:620227. doi: 10.3389/fmicb.2021.620227 (PMC8062866; doi:10.3389/fmicb.2021.620227)

## Supplementary Material

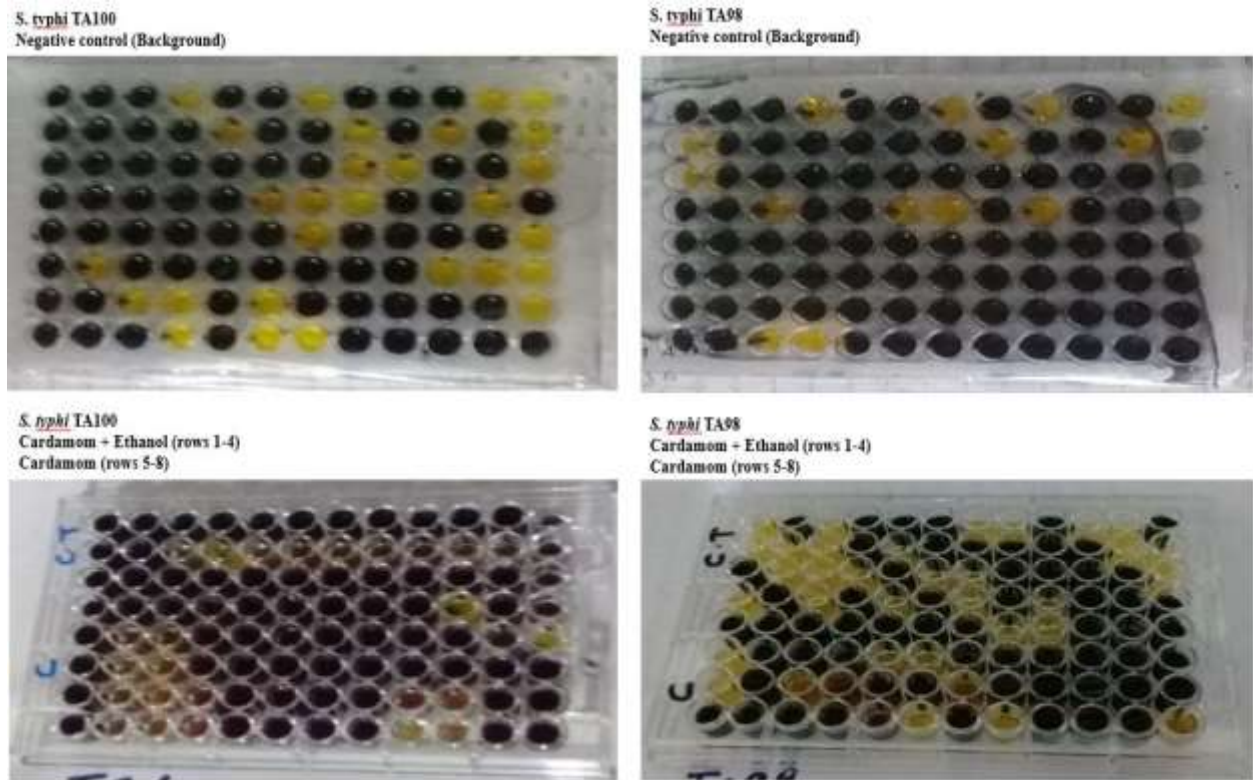

**Figure S1.** Mutagenicity assay of GCEO along with negative control.

Supplement: Supplementary file 1 [file Image_1.pdf]
